# Comparison of Clinical and Radiological Outcomes Between Uncemented and Cement Augmented Screws in Short Segment Hybrid Fixation of Unstable Osteoporotic Vertebral Fractures

**DOI:** 10.3390/jcm15041414

**Published:** 2026-02-11

**Authors:** Josef Vcelak, Adam Kral, Lucie Sedova, Jan Lesenky, Ondrej Seda

**Affiliations:** 1 Department of Orthopaedics, First Faculty of Medicine, Charles University and Bulovka University Hospital, 180 81 Prague, Czech Republic; 2 Institute of Biology and Medical Genetics, First Faculty of Medicine, Charles University and General Teaching Hospital, 128 00 Prague, Czech Republic

**Keywords:** hybrid fixation, osteoporotic vertebral fracture, cement screw augmentation, vertebroplasty, transpedicular fixation

## Abstract

**Purpose:** This study aimed to compare the clinical and radiological outcomes of treating unstable osteoporotic vertebral fractures using hybrid fixation with uncemented transpedicular screws (Group A) versus polymethylmethacrylate (PMMA) cement-augmented screws (Group B). **Methods**: A retrospective comparative study of 55 patients treated between 01/2017 and 03/2024. Group A included 35 patients (mean age 71.22 ± 6.12 years); Group B included 20 patients (mean age 72.9 ± 7.75 years). Clinical outcomes were compared preoperatively, 6 weeks and 1 year after surgery. For clinical evaluation, the ODI and VAS for back pain were used. Restoration of the sagittal spinal profile was evaluated using the sagittal Cobb angle and the height of the fractured vertebral body. **Results**: Both groups showed significant clinical improvement in ODI and VAS scores at 6 weeks and 1 year postoperatively (*p* < 0.001), without significant between-group differences. The ODI changed from a preoperative value of 75.07 ± 21.13 to 50.72 ± 17.7 at 6 weeks postoperatively, and to 28.83 ± 20.9 at 1 year postoperatively in Group A. In Group B, the preoperative ODI value of 66.8 ± 14.56 changed to 47.00 ± 11.72 at 6 weeks and to 19.8 ± 9.15 at 1 year postoperatively The VAS decreased from 7.55 ± 1.43 preoperatively to 3.50 ± 1.17 at 6 weeks and to 1.52 ± 1.18 at 1 year postoperatively in Group A, and from 7.53 ± 1.39 preoperatively to 2.53 ± 1.02 at 6 weeks and to 1.52 ± 1.18 at 1 year postoperatively in Group B. In Group A, preoperative Cobb angle values of 11.01 ± 13.85 improved to 7.33 ± 16.17 at 6 weeks, with subsequent loss to 12.96 ± 14.75 degrees at 1 year postoperatively. In Group B, preoperative values were 11.44 ± 17.84, corrected to 5.16 ± 8.33 at 6 weeks, and to 7.37 ± 9.28 degrees at 1 year postoperatively. **Conclusions:** Good clinical outcomes were achieved in both evaluated groups using uncemented or cement-augmented screws, without a statistically significant difference. Differences were noted in the radiological evaluation of the success of sagittal profile correction. While both groups showed initial radiological improvement, the uncemented screw group experienced a statistically significant loss of correction at the 1-year follow-up.

## 1. Introduction

Hybrid fixation is a widely accepted surgical treatment method for unstable osteoporotic vertebral fractures (OVF) [1]. The indications for this treatment include fractures of the thoracolumbar spine involving one or both endplates and more than one-fifth of the posterior wall of the vertebral body, classified according to osteoporotic classification (OF classification) [2] as types 3 and 4 [3,4]. This technique may also be considered for fractures exhibiting an “intervertebral cleft” or vertebral body osteonecrosis [5]. The combination of the cement augmentation of the fractured vertebral body and short posterior transpedicular instrumentation functionally simulates a 360-degree reconstruction of the spine, restoring stability and preventing further collapse of the posterior vertebral wall [6,7]. An undeniable advantage is the possibility of restoring the sagittal spinal profile with a relatively low surgical burden in often polymorbid elderly patients. However, a disadvantage is the risk of construct failure due to screw migration in the osteoporotic bone, most commonly via the windshield-wiper mechanism, leading to loss of the initially achieved correction and recurrence of instability in the treated segment [8,9,10]. Another risk is perioperative cement leakage from the vertebral body during vertebroplasty (VP) or kyphoplasty (KP) into the venous system, which can cause pulmonary embolism, or into the spinal canal, which can compress neural structures [11,12]. Various modifications to the surgical method have been introduced to prevent these frequent complications [13,14]. One of the most common modifications is the use of cement-augmented (CA) screws for posterior transpedicular fixation [15]. Cementing screws using the “chemical anchor” principle increases the stability of the pedicle screw within the vertebral body, specifically at the point of least resistance of the posterior instrumentation during cyclic loading at the screw–bone interface. Conversely, this method may be burdened with a higher risk of cement leakage, infection followed by complicated revision surgery, and increased risk of junctional failure manifested as adjacent vertebral fracture due to the elevated rigidity of the construct [16]. The balancing of these risks continues to fuel the debate on whether to use cemented transpedicular fixation or uncemented screws with modifications to the screw design or construct geometry.

This study aimed to compare the clinical and radiological outcomes of treating unstable OVF using hybrid fixation with uncemented transpedicular screws in one group of patients and CA screws in another group. We formulated two hypotheses: (1) clinical outcomes would be significantly better in patients treated with hybrid fixation using cement-augmented screws, and (2) patients treated with uncemented screws would demonstrate a higher risk of correction loss and instrumentation failure based on radiological assessment.

## 2. Materials and Methods

A single-center retrospective comparative study assessing the clinical and radiological results of patients treated with hybrid fixation of unstable osteoporotic fractures using uncemented and CA screws was conducted from January 2017 to March 2024 at a university hospital (tertiary center). All patients provided written informed consent upon admission, and the data were retrospectively collected from medical records. The study was approved by the hospital’s institutional ethics committee.

During the evaluation period, a total of 35 patients (men: 4, women: 31, average age 71.22 ± 6.12) were treated in Group A using hybrid fixation with uncemented screws. In Group B, 20 patients (men: 0, women: 20, average age 72.9 ± 7.75) were treated using CA screws for fixation. The development of the hybrid fixation technique, aimed at increasing construct stability through the introduction of PMMA-cemented screws, at our institution resulted in the formation of a second patient group. As this technique was introduced later and adequate postoperative follow-up was required, the sample sizes of the non-cemented and cemented screw groups were asymmetric, which was taken into account in the statistical power analysis. The indication for hybrid fixation was an unstable OVF of the thoracolumbar spine classified as OF type 3 or 4. Inclusion criteria were defined as follows: 1. age over 60 years; 2. confirmed osteoporosis (DEXA, densitoCT, densitoUS); 3. clinical findings (axial pain, neurologic symptomatology); 4. acute fracture (within 3 months of symptom onset); 5. normal neurological status ASIA E; 6. single-level fracture. Conversely, the exclusion criteria for the study were defined as: 1. age under 60 years; 2. a different fracture pathology; 3. chronic post-traumatic deformity; 4. persistent neurological deficit; 5. ASA (American Society of Anesthesiologists) risk IV, V. Spinal tissue sampling with histopathological analysis was performed in every case to exclude any metastatic origin of the fracture. Table 1 summarizes the basic demographic data of both patient groups.

All patients underwent open hybrid stabilization of the fracture, including short transpedicular fixation one level above and below the fractured vertebra with its reduction by ligamentotaxis, followed by filling of the affected vertebral body with cement using the VP technique (Vertecem V+ Cement Kit, Depuy-Synthes, West Chester, Pennsylvania). The difference between the two evaluated groups was the technique of transpedicular screw insertion. In Group A, polyaxial screws were inserted using the classical method, while in Group B, cement augmentation of the screws was used. The insertion channel for the screws in Group B was primarily prepared using transpedicular introducers and pre-drills, ensuring no perforation in the area of the pedicle. A Jamshidi needle was then introduced into the channel, and the channel was subsequently injected with cement at the junction of the anterior and middle thirds of the vertebral body, followed by screw insertion. Each surgical step was controlled using lateral-view fluoroscopy to minimize the risk of cement leakage. In the postoperative period, patients were mobilized from the first postoperative day using verticalization aids.

Clinical outcomes were compared preoperatively, 6 weeks postoperatively, and 1 year postoperatively. For clinical evaluation, the Oswestry Disability Index (ODI) (0–100 points) and the Visual Analog Scale (VAS) for back pain were used. Additionally, the necessity of revision surgery due to complications within the 1-year postoperative interval (neurological deterioration requiring open decompression, superficial infection, deep infection) was recorded.

Radiological evaluation was performed by X-ray assessment preoperatively, at 6 weeks and at 1 year after surgery. Restoration of the sagittal spinal profile was evaluated using the modified sagittal Cobb angle. The kyphosis angle was measured between the plane of the superior endplate of the upper instrumented vertebra and the inferior endplate of the lower instrumented vertebra. The height of the fractured vertebral body was also measured. Owing to the asymmetry of the vertebral body deformation, the heights of the anterior, central, and posterior vertebral walls were measured according to the methodology of Spiegl [9]. In the postoperative radiological evaluation, cement leakage from the fractured vertebral body treated by VP was also recorded in both evaluated groups, and leakage from CA screws was recorded in Group B. Additionally, during the evaluated time interval, the manifestation of adjacent vertebral fractures and instrumentation failure (windshield-wiper effect, screw radiolucency, screw pull-out) was recorded within the 1-year postoperative period.

A *p* value < 0.05 was considered significant. Statistical analysis was performed using Student’s paired *t*-test to evaluate differences between preoperative and postoperative measurements in each assessed group. An unpaired *t*-test (statistically significant difference between measurements *p* < 0.05) was used for subsequent comparisons to determine the decisive mutual difference between specific pairs (Group A vs. Group B).

## 3. Results

In Group A, 35 patients were initially evaluated clinically and radiologically preoperatively and at 6 weeks postoperatively. At the 1-year postoperative interval, 31 patients were evaluated (four patients died between 6 weeks and 1 year after surgery, without direct relation to the operative procedure, and were therefore not included in the 1-year statistical analysis.). Among clinical complications, one case of superficial infection and two cases of deep infection requiring surgical revision with retention of posterior instrumentation were recorded. No other early postoperative clinical complications were observed. In Group B, a total of 20 patients were evaluated preoperatively, then at 6 weeks and at 1 year postoperatively. Only one case of early revision for superficial infection requiring revision with the posterior instrumentation retain was recorded in this group.

**Figure 1:**
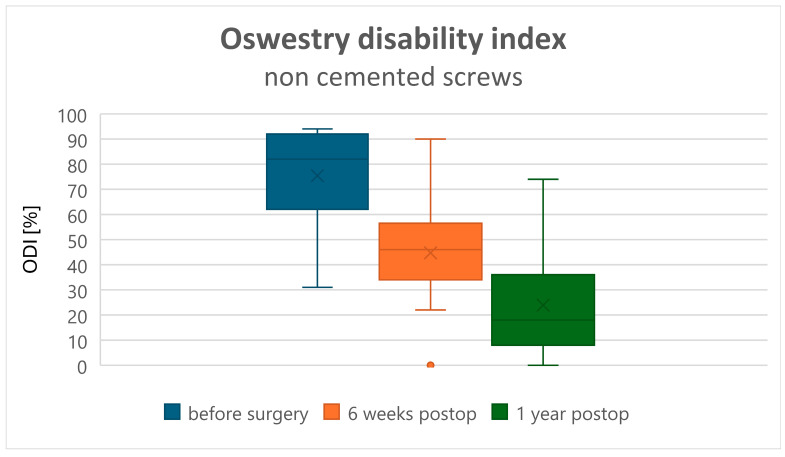
Oswestry Disability Index (ODI) scores in the uncemented screw group.

**Figure 2:**
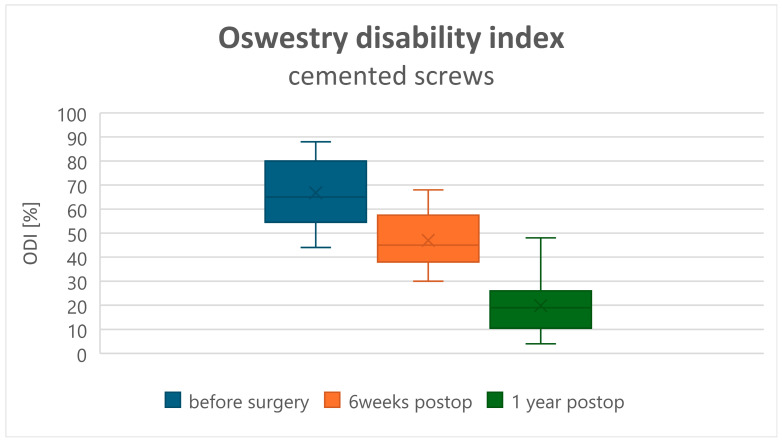
Oswestry Disability Index (ODI) scores in the cemented screw group.

**Figure 3:**
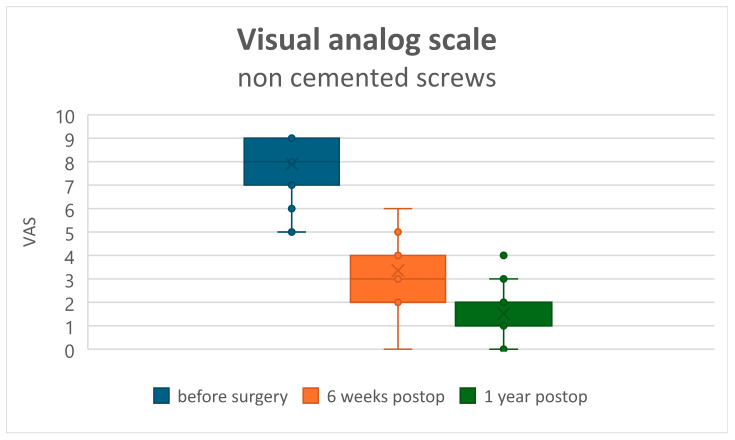
Visual Analog Scale (VAS) for back pain in the uncemented screw group.

**Figure 4:**
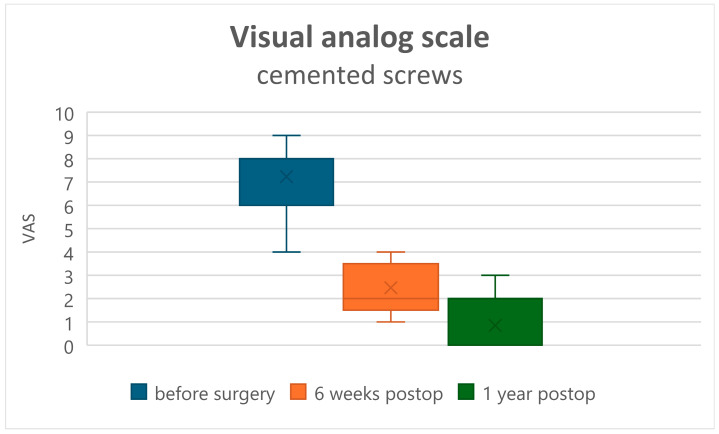
Visual Analog Scale (VAS) for back pain in the cemented screw group.

Both clinical assessments, ODI and VAS, improved in all patients at 6 weeks and 12 months postoperatively (*p* < 0.001). The recovery rate assessed by the ODI changed from a preoperative value of 75.07 ± 21.13 to 50.72 ± 17.7 at 6 weeks postoperatively, and to 28.83 ± 20.9 at 1 year postoperatively in Group A (Figure 1). In Group B, the preoperative ODI value of 66.8 ± 14.56 changed to 47.00 ± 11.72 at 6 weeks and to 19.8 ± 9.15 at 1 year postoperatively (Figure 2). Comparison of the ODI value decrease, and thus the clinical improvement, showed no statistically significant difference between Groups A and B (Table 2). VAS (back pain) decreased from 7.55 ± 1.43 preoperatively to 3.50 ± 1.17 at 6 weeks and to 1.52 ± 1.18 at 1 year postoperatively in Group A (Figure 3), and from 7.53 ± 1.39 preoperatively to 2.53 ± 1.02 at 6 weeks and to 1.52 ± 1.18 at 1 year postoperatively in Group B (Figure 4). Comparison of VAS value regression, and thus pain reduction, between the two groups showed no statistically significant difference (Table 3).

**Figure 5:**
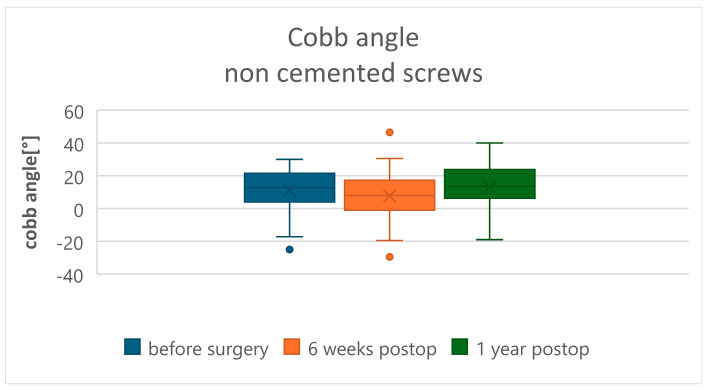
Modified Cobb angle in the uncemented screw group.

**Figure 6:**
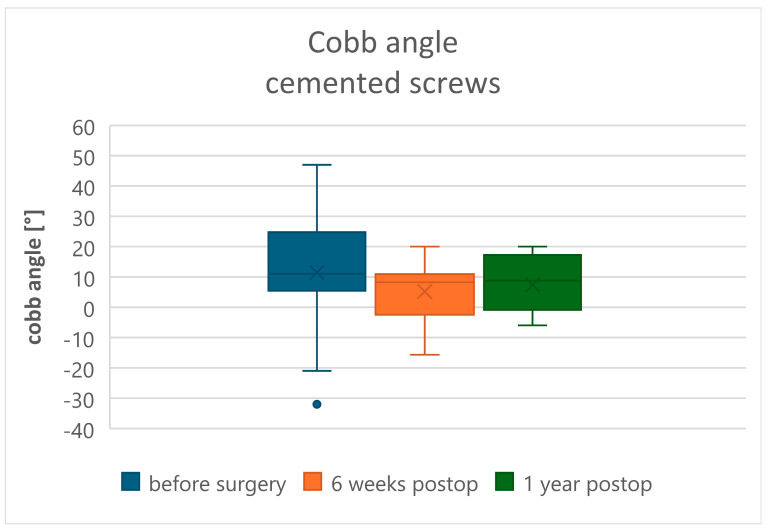
Modified Cobb angle in the cemented screw group.

**Figure 7:**
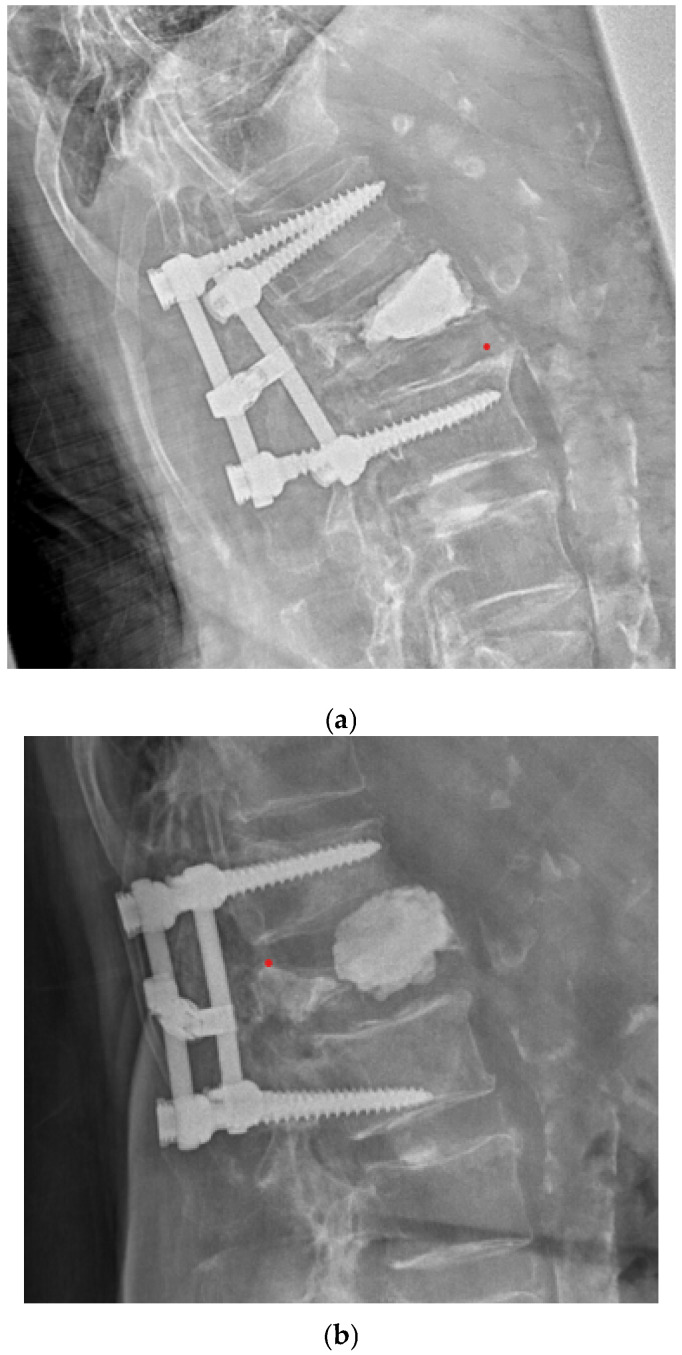
(**a**,**b**) Lateral radiograph of a patient from the uncemented screw group (Group A) demonstrating an intervertebral cleft with anterior displacement of a vertebral body fragment, associated with a centrally located cement block from vertebroplasty.

In the radiological evaluation, measurement of the modified Cobb angle demonstrated statistically significant kyphosis reduction and sagittal profile correction in both Groups A and B at 6 weeks postoperatively, followed by loss of correction at 1 year after surgery (*p* < 0.05). In Group A, preoperative modified Cobb angle values of 11.01 ± 13.85 degrees improved to 7.33 ± 16.17 degrees at 6 weeks, with subsequent loss to 12.96 ± 14.75 degrees at 1 year postoperatively (Figure 5). In Group B (CA screws), preoperative values were 11.44 ± 17.84 degrees, corrected to 5.16 ± 8.33 degrees at 6 weeks, and to 7.37 ± 9.28 degrees at 1 year postoperatively (Figure 6). At the 1-year postoperative evaluation, a statistically significant decrease in reduction (*p* = 0.01) was observed in Group A (uncemented screws) compared to Group B (CA screws) (Table 4). The evaluation of vertebral body height correction (anterior, central, and posterior wall) is clearly presented in Table 5. In both groups, there was a statistically significant correction of vertebral body height in the early postoperative period. During the 1-year follow-up, a difference was noted between the groups: in Group B (cemented screws), the reduction in vertebral height loss was significantly less compared to Group A. During the evaluation period, instrumentation failure due to screw loosening (clear zone) or the windshield-wiper effect (the methodology described by Sanden et al. [17]) was observed in 10 patients (32.26%) in Group A, necessitating two revision surgeries within the follow-up period. In both cases, the instrumentation was extended proximodistally by one more spinal segment. Additionally, in Group A, one patient presented with an intervertebral “cleft” causing displacement of the anterior portion of the vertebral body due to migration of the distal fixation screws, resulting in loosening and axial rotation of the vertebral body cement block (Figure 7a,b). In Group B, one case (5%) of posterior instrumentation failure requiring revision by extending the fixation was observed.

Cement leakage from the vertebra was recorded in 13 patients (37.14%) in Group A (7× into the intervertebral disc, 1× into the spinal canal, 5× into a segmental vein) without clinical symptomatology and without need for revision surgery. In Group B, cement leakage was observed in 8 patients (40%) (6× into the intervertebral disc, 1× into the spinal canal, 1× into a segmental vein). In the patient with cement leakage into the spinal canal, decompressive surgery was performed immediately without clinical manifestation of neurological deficit. Additionally, within the 1-year postoperative interval, adjacent vertebral fractures were recorded in three patients in Group A (9.7%) and two patients in Group B (10%). In all these cases, vertebroplasty of the fractured vertebra was performed as a second-stage procedure.

## 4. Discussion

Hybrid fixation of unstable OVF currently represents a surgical treatment option with the advantages of minimally invasive management. An undeniable advantage is the short extent of fixation, a minimally invasive alternative for dorsal instrumentation, and the possibility of correcting post-traumatic kyphosis with subsequent restoration of the stability of the anterior spinal column without the necessity for anterior surgery. Conversely, a disadvantage may be the risk of posterior instrumentation failure, resulting in loss of reduction and the development of instability. As a result, the method has been repeatedly modified. The present study compared the results between groups using uncemented and cement-augmented screws for posterior instrumentation.

The first hypothesis, testing the effect of clinical outcomes in patients treated with hybrid fixation using uncemented and CA screws, was not confirmed. In both groups, significant clinical improvement and pain relief were achieved within the evaluated interval, without a statistically significant difference. No severe acute complications that negatively affected the final evaluation were recorded. No serious neurological deficits or patient deterioration associated with the treatment were observed in either group. Revision surgeries in the early postoperative period were performed only due to wound healing disorders or infectious complications. Despite early mobilization and resumption of daily activities, mortality was recorded in four patients (7.3%) within the 1-year follow-up, without any direct association with the surgery itself. Clinical improvement has also been achieved by other authors using various methods to treat unstable OVF. Vougioukas [12] combined vertebroplasty and short instrumentation with minimally invasive cannulated cemented screws as part of hybrid fixation. Among all nine patients, significant clinical improvement was achieved without major complications, although statistically significant loss of initially achieved correction was observed in eight cases. Rong [18], in a series of 28 patients treated with fracture stabilization using CA screws without vertebral body augmentation, achieved clinical improvement (ODI from 57.39% to 6.83%) with asymptomatic cement leakage in 10.3% of the patients. Hu [19] compared treatment of osteoporotic fractures using classical cementing through Jamshidi needles versus cannulated screws. Clinical evaluation showed an ODI score improvement from 76.5% to 40.2% in the first group and from 71% to 28% in the second group, with comparable VAS improvements in both groups. Octenoglu [20] combined posterior instrumentation with kyphoplasty and, despite significant kyphosis progression postoperatively, achieved marked clinical improvement. Mahmoud [8] reported that clinical outcomes were significantly better in lumbar fractures compared to thoracic and thoracolumbar spine fractures. In contrast, Spiegl [9], in a group of 113 patients treated with hybrid fixation over a 2-year period, observed 15% mortality and serious complications in five patients (4.4%) (one case of cauda equina syndrome, three cases of wound healing disorders, one pneumonia).

The second hypothesis, which assessed the radiological outcomes of maintaining the achieved vertebral body height and sagittal profile reduction, was confirmed. In the group of patients where uncemented transpedicular screws were used, a statistically significant loss of correction was recorded within the 1-year postoperative interval. Radiological evaluation showed a decrease in the anterior vertebral wall height and a reduction of the modified Cobb angle, resulting in kyphosis progression in the treated segment. Signs of fixation loosening at the screw–bone interface (clear zone) or construct migration due to the windshield-wiper effect were also noted in 32% of patients, mostly without clinical correlation. Only two patients required revision surgery during the follow-up period due to confirmed fixation failure, which necessitated the extension of dorsal instrumentation and resulted in healing. The need for revision surgery in some patients in the uncemented screw group could potentially distort the overall clinical assessment at the 1-year evaluation for this group, though clinical outcomes were statistically similar. Only one patient was diagnosed with an intervertebral cleft of the vertebral body. The reason for the complication was the formation of a cement block localized centrally in a biconcave fracture of the vertebral body. Subsequent axial loading led to the separation of the ventral third of the vertebral body anteriorly. The patient was treated conservatively with a Jewett brace and achieved healing without the need for revision surgery. The mechanism of correction loss due to screw loosening in osteoporotic bone is a widely discussed issue. Spiegl [7] attempted to identify the anatomical structures responsible for the loss of achieved correction (kyphosis progression). In a group of 29 patients evaluated two years postoperatively, although no implant failure was noted, a significant reduction in the height of the central vertebral body and upper adjacent disc was found. Weiser [10] compared the stability of short fixation with CA screws to long instrumentation. In a cadaveric in vitro study on 12 specimens, he assessed a 16% increase in screw stability with CA screws and a 76% increase with long 2 + 2 segment fixations during cyclic loading but found no significant difference in the number of cycles to failure between the groups. Coniglio [21] extended the instrumentation to 2 + 2 segments using CA screws to prevent subsequent kyphosis progression. He noted a minimal loss of correction of two degrees of vertebral body kyphosis in the postoperative period and clinically insignificant cement leakage in 11% of patients. Spiegl [22] compared long hybrid instrumentation with the cementing of all screws versus cementing only the end screws. He found no difference in failure rates between the two setups in a cadaveric quasi-static loading study, with significantly lower lever arms observed with full cementation.

Cement leakage was observed in both evaluated groups. In the group with uncemented screws, leakage was present in 37% of cases, most commonly as extravasation into the adjacent upper intervertebral disc, without any clinical correlation. In the group of patients treated with CA screws, cement leakage was diagnosed in 40% of cases. Adding screw cementation did not substantially increase the overall patient cement leakage rate beyond that already seen with VP. In both groups, cement leakage into the venous system was not associated with subsequent pulmonary embolism. Cement leakage into the spinal canal in the CA screw group necessitated decompressive surgery during the same procedure, without clinical manifestation of neurological deficit. Generally, cement leakage from cemented screws has been reported in 20–93% of patients [23]. Hu [19], using the classical cementing technique, reported cement leakage in 17.9% of cases, with 11.7% leaking into the spinal canal, and statistically insignificantly lower leakage rates (13.6%) with cannulated screws, of which 8% leaked into the spinal canal without clinical correlates. He identified higher screw stability with cannulated screws with multiple holes, but a higher risk of cement leakage into the spinal canal from holes located in the proximal threaded part of the screw. Dai [24], in his study, did not report clinically significant cement leakage. He used a technique with cannulated screws having three holes in the threaded part, achieving filling without creating significant injection pressure. He pointed out the risk of screw failure in cannulated screws with holes at the apex of the screws. Subsequent loosening of screws at the pedicle isthmus increases the risk of fatigue fracture of the screw. He also emphasized the risks associated with revision surgeries requiring screw removal. Mengis [25] similarly points out the risk of revision surgeries for infections when using augmented screws.

Junctional failure of hybrid fixation manifested by adjacent vertebral fractures was recorded equally in both groups. In all cases, the proximal vertebra was involved. The development of this complication was not associated with the manifestation of neurological deficits. In all cases, treatment involved supplementary vertebroplasty of the fractured vertebra as a two-stage revision. Spiegl [9] found manifestation of a new adjacent fracture in 17.4% of cases, with an average time to manifestation of 6.9 months postoperatively. Aboud [26], in a cohort of 165 patients, compared the occurrence of adjacent segment fractures between vertebral augmentation alone and hybrid transpedicular stabilization. He found a significantly higher incidence in patients treated with vertebral cement augmentation (26%) than in those treated with hybrid fixation (11%), with a median time to manifestation of 10.9 weeks after the primary operation. Other authors [27,28] defined the so-called “transition zone” between the relatively rigid segment stabilized by fixation and the periphery of the instrumentation, the so-called “soft landing concept”, with an up to one-third increased risk of junctional failure. In osteoporotic spines, this most commonly manifests not in a disc but as a pathological fracture of the adjacent segment [29], usually proximally. The risk increases with a larger volume of cement installed or leakage into the disc space.

This study compared the clinical and radiological outcomes of two modified methods of hybrid fixation for osteoporotic spinal fractures. Considering the demographic aging of the population and the increasing incidence of this problem, the new findings of this study contribute to the development of surgical treatment for unstable OVF. The evaluated patient cohort was homogeneous, consisting of predominantly women aged over 60 years, with the exclusion of fractures of other etiologies. The surgical approach in both evaluated groups was identical, with the only difference being the use of cement augmentation of the screws in the short posterior instrumentation. This single evaluated variable in the surgical procedure enhances the relevance of the clinical and radiological results. However, this study has certain limitations. The main limitation of this study is its retrospective design and the statistically limited number of patients. A prospective study with a larger cohort would provide more robust evidence regarding the comparative efficacy of these techniques.

## 5. Conclusions

Hybrid fixation is a widely accepted surgical method for the treatment of unstable OVF. In our cohort of patients treated by the method, no lasting severe clinical complications associated with cement leakage from the vertebral body or during screw augmentation were observed. Good clinical outcomes were achieved in both evaluated groups using uncemented or cement-augmented screws, without a statistically significant difference between the groups. Differences were noted in the radiological evaluation of the success of sagittal profile correction, as measured by the modified Cobb angle and the vertebral body height. The initially significant improvement in reduction parameters in both groups, evaluated 6 weeks postoperatively, was statistically significantly worsened at the 1-year postoperative follow-up in the group with uncemented screws, whereas in the CA screw group, the decline was statistically insignificant.

## Figures and Tables

**Table 1:** Overview of the basic demographic data of both patient groups.

	Non Cemented Screws	Cemented Screws
Age	71.22 ± 6.12	72.9 ± 7.75
Sex (male/female)	4/31	0/20
Vertebral fractures	38	25
Anatomical localisation		
T6	1	0
T7	1	0
T8	1	0
T9	1	0
T10	1	0
T11	1	4
T12	9	7
L1	14	9
L2	5	2
L3	3	2
L4	1	1
L5	0	0
OF classification		
I	0	0
II	2	0
III	25	15
IV	8	5
ASA score		
I	0	0
II	2	0
III	20	12
IV	13	8

**Table 2:** The mean Oswestry Disability Index (ODI) scores (±standard deviation) for the uncemented screw group (Group A) and the cemented screw group (Group B) at preoperative assessment, 6 weeks postoperative, and 1 year postoperative.

Oswestry Disability Index (ODI)							
	Before Surgery	6 Weeks Postop	Difference	*p*-Value	1 Year Postop	Difference	*p*-Value
**non-cemented screws**	75.07 ± 21.13	50.72 ± 17.7	24.34 ± 15.96	*p* < 0.0001	28.83 ± 20.9	21.9 ± 13.6	*p* < 0.0001
**cemented screws**	66.8 ± 14.56	47.00 ± 11.72	19.8 ± 11.46	*p* < 0.0001	19.8 ± 9.15	27.2 ± 9.37	*p* < 0.0001

**Table 3:** The mean Visual Analog Scale (VAS) scores for back pain (±standard deviation) for the uncemented screw group (Group A) and the cemented screw group (Group B) at preoperative assessment, 6 weeks postoperative, and 1 year postoperative.

Visual Analog Scale (VAS)							
	Before Surgery	6 Weeks Postop	Difference	*p*-Value	1 Year Postop	Difference	*p*-Value
**non-cemented screws**	7.55 ± 1.43	3.50 ± 1.17	4.26 ± 1.36	*p* < 0.0001	1.52 ± 1.18	1.97 ± 0.91	*p* < 0.0001
**cemented screws**	7.53 ± 1.39	2.53 ± 1.02	5.00 ± 1.24	*p* < 0.0001	0.9 ± 1.15	1.63 ± 0.96	*p* < 0.0001

**Table 4:** The mean modified Cobb angles in degrees (°) (±standard deviation) for the uncemented screw group (Group A) and the cemented screw group (Group B) at preoperative assessment, 6 weeks postoperative, and 1 year postoperatively.

Cobb Angle							
	Before Surgery [°]	6 Weeks Postop [°]	Difference [°]	*p*-Value	1 Year Postop [°]	Difference [°]	*p*-Value
**non-cemented screws**	11.01 ± 13.85	7.33 ± 16.17	3.28 ± 9.47	*p* = 0.03	12.96 ± 14.75	−5.32 ± 9.16	*p* = 0.002
**cemented screws**	11.44 ± 17.84	5.16 ± 8.33	6.29 ± 15.34	*p* = 0.04	7.37 ± 9.28	−2.22 ± 9.21	*p* = 0.02

**Table 5:** The mean heights (in mm, ±standard deviation) of the anterior, middle, and posterior columns of the fractured vertebral body for both the uncemented screw Group A (Group A, ‘non cemented screws’) and the cemented screw group (Group B, ‘cemented screws’) at preoperative, 6 weeks postoperative, and 1-year postoperative assessments.

**Non-Cemented Screws**				
	**Before Surgery [mm]**	**6 Weeks Postop [mm]**	**1 Year Postop [mm]**	***p*-Value**
**anterior column**	13.36 ± 5.4	22.5 ± 6.92	20.10 ± 5.87	*p* < 0.00001
**middle column**	10.75 ± 4.24	21.69 ± 6.5	19.66 ± 6.72	*p* < 0.00001
**posterior column**	21.91 ± 3.9	26.80 ± 5.75	25.54 ± 5.36	*p* < 0.00001
**Cemented Screws**				
	**Before Surgery [mm]**	**6 Weeks Postop [mm]**	**1 Year Postop [mm]**	***p*-Value**
**anterior column**	14.12 ± 6.45	19.19 ± 6.5	18.20 ± 5.92	*p* < 0.00001
**middle column**	10.74 ± 4.53	17.72 ± 6.38	17.29 ± 6.32	*p* < 0.00001
**posterior column**	22.38 ± 3.08	23.38 ± 3.68	23.47 ± 3.55	*p* = 0.01
	**Difference Before Surgery–6 Weeks Postop**		**Difference 6 Weeks Postop–1 Year Postop**	
**anterior column**	9.13 ± 5.20		1.92 ± 3.59	
**middle column**	10.94 ± 6.59		2.03 ± 3.59	
**posterior column**	4.89 ± 4.28		1.26 ± 2.45	
	**Difference Before Surgery–6 Weeks Postop**		**Difference 6 Weeks Postop–1 Year Postop**	
**anterior column**	5.07 ± 4.69		0.99 ± 2.18	
**middle column**	6.98 ± 5.32		0.44 ± 0.88	
**posterior column**	1.50 ± 2.53		0.40 ± 0.97	
	***p*(ant) = 0.003**		***p*(ant) = 0.06**	
	***p*(midl) = 0.005**		***p*(midl) = 0.02**	
	***p*(post) = 0.001**		***p*(post) = 0.07**	

## Data Availability

The datasets generated during and/or analyzed during the current study are available from the corresponding author upon a reasonable request.
